# Supplementary material for: Acinetobacter baumannii under Acidic Conditions Induces Colistin Resistance through PmrAB Activation and Lipid A Modification
Source: Antibiotics (Basel). 2023 Apr 26;12(5):813. doi: 10.3390/antibiotics12050813 (PMC10215926; doi:10.3390/antibiotics12050813)
Supplement: Supplementary file 1 [file antibiotics-12-00813-s001.zip › antibiotics-2352836-supplementary.pdf]

antibiotics

Article

Acinetobacter baumannii under Acidic Conditions Induces Colistin Resistance through PmrAB Activation and Lipid A Modification

Supplementary Materials

Construction of ΔpmrA and ΔpmrB mutant strains in A. baumannii ATCC 17978

The deletion mutants of Δ*A1S_2751* (*pmrA*) and Δ*A1S_2750* (*pmrB*) in *A.baumannii* ATCC 17978 were constructed using markerless gene-deletion method (Supplementary Figure S1A and S1B). The upstream and downstream regions of *A1S_2751* were amplified using the primer sets of 2751_Up_SpeI (F)/2751_Up (R), and 2751_Down (F)/2751_Down (R), respectively (Supplementary Table S1). The upstream and downstream regions of *A1S_2750* were amplified using the primer sets of 2750_Up_SpeI (F)/2750_Up (R), and 2750_Down (F)/2750_Down (R), respectively. The *nptI*, conferring resistance to kanamycin, was amplified using the primer sets of 2751_ NptI (F)/NptI_ApaI (R), and 2750_ NptI (F)/NptI_ApaI (R). The three PCR products consisting of upstrem and downstream regions of target gene and *nptI* were assembled by overlap extension PCR using the 2751_Up_SpeI (F)/NptI_ApaI (R) primers for the Δ*pmrA* mutant, and 2750_Up_SpeI (F)/NptI_ApaI (R) primers for the Δ*pmrB* mutant. The assembled DNA fragments and pDM4 plasmid vector were double-digested using *Spe*I (Enzynomics, Korea) and *Apa*I (Takara, Japan) restriction enzymes, and then ligated using T4 DNA ligase (Toyobo, Japan). The constructed plasmids were transformed to *E. coli* DH5α by heat-shock method. The colonies containing the constructed plasmids were selected on LB plates containing kanamycin and chloramphenicol. The plasmids were harvested using a plasmid mini-prep kit (GeneAll, Korea) and the exact sequences were analyzed (Macrogen, Korea). The purified plasmids were transformed to the conjugal donor *E. coli* SM10, and then transferred to *A. baumannii* ATCC 17978 by conjugation. The mutnat strains that accomplished cross-over homologous-recombination were selected on LB plates containing kanamycin as the first selection, and then selected on the LB plates containig 10% sucrose. Deletion of the genes was confirmed by PCR analysis using 2750_Up_SpeI (F) and 2751_Down (R) primers (Supplementary Figure S1D). The Δ*pmrA* and Δ*pmrB* mutants were named HJ2751D and HJ2750D, respectively (Table 1).

Construction of pmrA-complemented A. baumannii strain

For the construction of the *pmrA*-complemented *A. baumannii* strain, the *pmrA* coding and *ompA* promoter regions were amplified using the primer sets of OmpA_2751 (F)/2751_T1 (R) and EcoRI_OmpA (F)/OmpA (R) using *A. baumannii* ATCC 17978 as a template (Supplementary Table S1). T1 terminator was amplified using the primer sets of 2751_T1 (F)/T1_PstI (R) using the pOH4 plasmid as a template. Three PCR products consisting of *ompA* promoter, *A1S_2751*, and T1 terminator were assembled by overlap extension PCR using the EcoRI_OmpA (F)/T1_PstI (R). The amplified PCR fragments were digested with *EcoR*I (Enzynomics, Korea) and *Pst*I (Enzynomics), and then introduced into the *EcoR*I and *Pst*I sites of pWH1266 plasmid with a source of a replication origin for *A. baumannii* (Supplementary Figure S1C). The constructed plasmids were transferred to *E. coli* DH5α by heat-shock method and the colonies were selected on LB plates containing tetracycline. The plasmids were purified using plasmid mini-prep kit and the sequences were analyzed. The constructed plasmids were transformed to HJ2751D strain by electroporation (Supplementary Figure S1D). The *pmrA*-complemented strain was named HJ2751C (Table 1).

Supplementary Table S1. Oligonucleotide primers used in this study

Primers	Sequence (5′ to 3′)
Construct for Δ*pmrA* and Δ*pmrB* mutants strains	
2751_Up_SpeI (F)	GGA CTA GTG CTC GAC CAT ACT TAA AGT TAC ATC
2751_Up (R)	GAG CCT AGA ACA TGT AAT TTA AAA TTT CGG GAC TTC ATA AAA GTG
2751_Down (F)	GTC CCG AAA TTT TAA ATT ACA TGT TCT AGG CTC GCT TAA G
2751_Down (R)	CGA GGC AGA CGA GCT CAT GCG GTA CG
2751_NptI (F)	CAT GAG CTC GTC TGC CTC GTG AAG AAG
2750_Up_speI (F)	GGA CTA GTC AAC AGG TCA TTT TTT CAA TCG G
2750_Up (R)	GGA CTT CAT AAA AGT GTG ATT ACG TAA GCT CTT GTT TCA
2750_Down (F)	GAG CTT ACG TAA TCA CAC TTT TAT GAA GTC CCG AAA TTT TAA ATT ATG
2750_Down (R)	GAG GCA GAC GGG TCA ATA ACG GTT TAG ATG G
2750_NptI (F)	CGT TAT TGA CCC GTC TGC CTC GTG AAG AAG
NptI_ApaI (R)	GTT GGG CCC GAT CCG TCGACC TGC AGG
Construct for *pmrA*-complemented strain	
EcoRI_OmpA (F)	GGA ATT CCG AGT GTT ATA GTG AGC TCA ACT G
OmpA (R)	TGG ATA TCC TCC AGA GAT AAC AAT TGT TG
OmpA_2751 (F)	ATC TCT GGA GGA TAT CCA TGA CAA AAA TCT TGA TGA TTG AAG ATG ATT TT
2751_T1 (R)	GTT TTA TTT GAT GCC TTA TGA TTG CCC CAA ACG G
2751_T1 (F)	GGG GCA ATC ATA AGG CAT CAA ATA AAA CGA AAG GC
T1_PstI (R)	GAA CTG CAG TCT AGG GCG GCG GAT TTG
Construct for plasmid with FLAG-tagged *pmrA*	
EcoRI_pmrAB_promoter (F)	GGA ATT CGT TCT CTG ATG ATG GTA CTG ATT ATG CG
Promoter_2751 (R)	CAT CAA GAT TTT TGT CAT AAA CAG GGA AAT CTG TTT ATT TCT TAA TG
Promoter_2751 (F)	CAG ATT TCC CTG TTT ATG ACA AAA ATC TTG ATG ATT GAA GAT G
FLAG_T1 (F)	GAC TAC AAA GAC GAT GAC GAC AAG TAA GCA TCA AAT AAA ACG AAA GGC TC
2751_FLAG (R)	GTC TTT GTA GTC TGA TTG CCC CAA ACG
pWH1266 (F)	GCA ACT TTA TCC GCC TCC ATC
pWH1266 (R)	GAT TTC ATA CAC GGT GCC TGA C

[*]Underlined sequences indicate regions that are not complementary to the templates.

Supplementary Table S2. Primers used in qPCR

Primers	Sequence (5′ to 3′)	Target genes	References
16S rRNA F	GCACAAGCGGTGGAGCAT	16S rRNA	This study
16S rRNA R	CGAAGGCACCAATCCATCTC		
PmrA F	GAGGTGGAATGGGTCAATAACG	*pmrA*	This study
PmrA R	GGCAATCCTAAATCCAAAAGAATAAG		
PmrB F	CTGTTGAGCAAGCGGGATTT	*pmrB*	This study
PmrB R	CTGACTGACCTGAATCTGATAATCCTT		
PmrC F	CTGTTGAGCAAGCGGGATTT	*pmrC*	This study
PmrC R	CTGACTGACCTGAATCTGATAATCCTT		

(A)

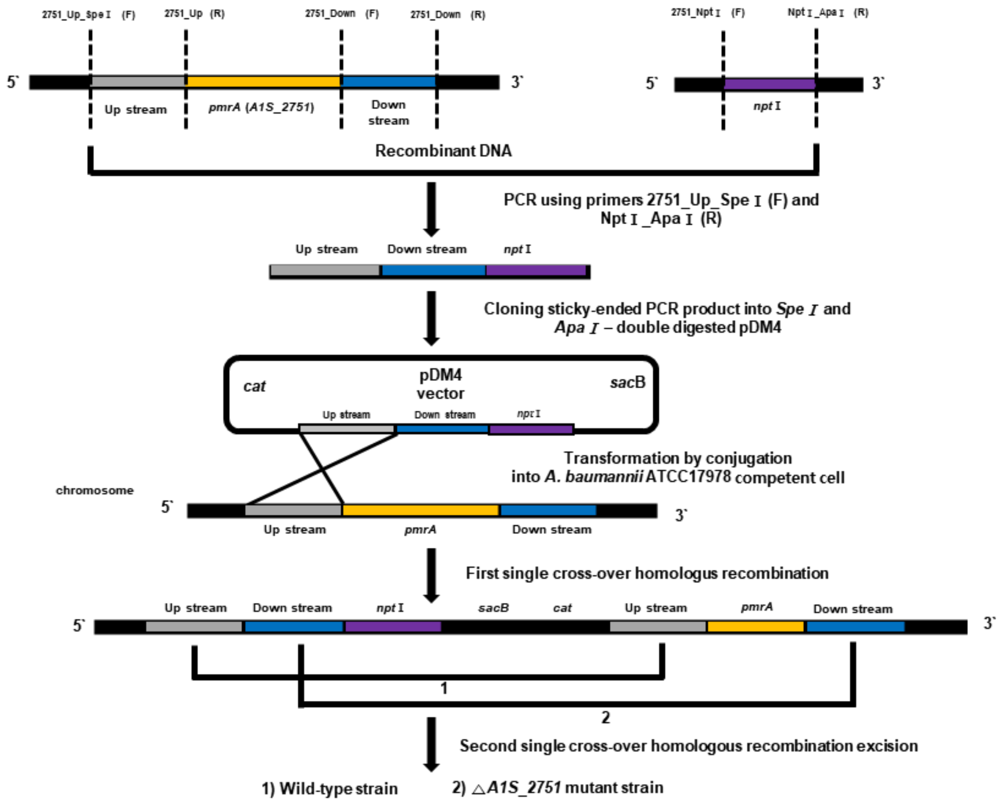

2751_Up_SpeⅠ (F)
2751_Up (R)
2751_Down (F)
2751_Down (R)
2751_NptⅠ (F)
NptⅠ_ApaⅠ (R)
5` 3`
Up stream
pmrA (A1S_2751)
Down stream
nptⅠ
Recombinant DNA
PCR using primers 2751_Up_SpeⅠ (F) and NptⅠ_ApaⅠ (R)
Up stream Down stream nptⅠ
Cloning sticky-ended PCR product into SpeⅠ and ApaⅠ – double digested pDM4
cat
pDM4 vector
sacB
Transformation by conjugation into A. baumannii ATCC17978 competent cell
chromosome
Up stream pmrA Down stream
First single cross-over homologus recombination
Up stream Down stream nptⅠ sacB cat Up stream pmrA Down stream
1
2
Second single cross-over homologous recombination excision
1) Wild-type strain
2) △A1S_2751 mutant strain

(B)

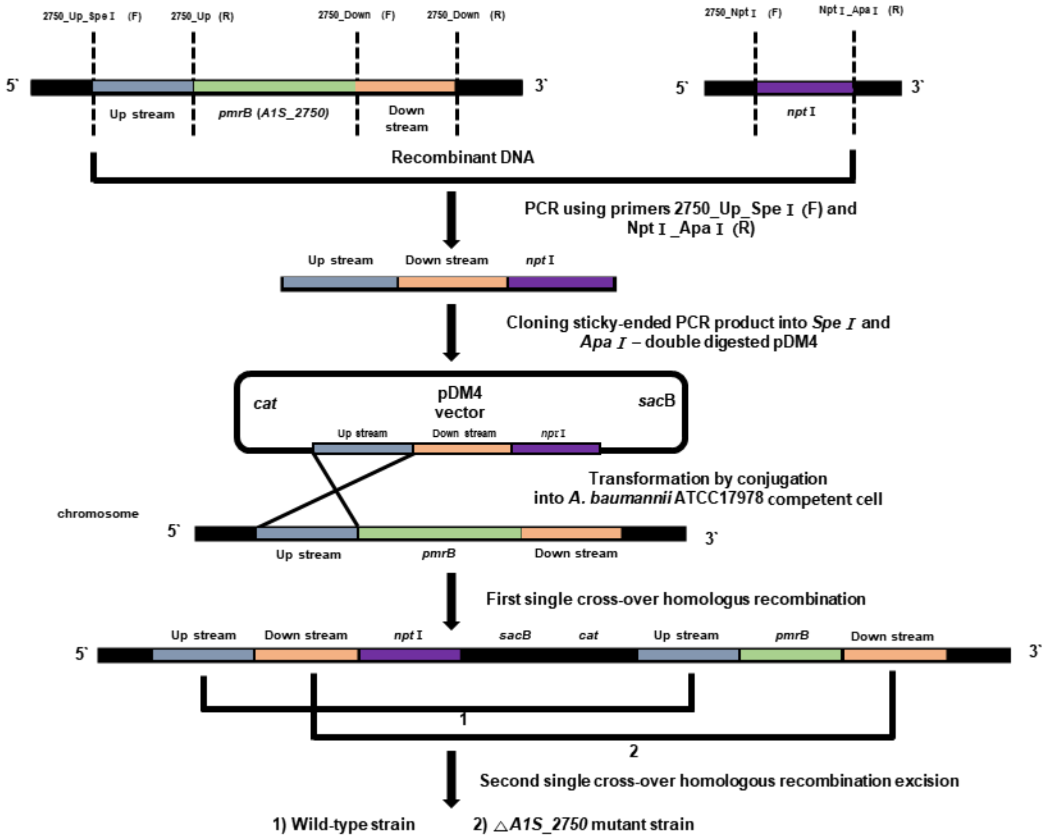

2750_Up_SpeⅠ (F)
2750_Up (R)
2750_Down (F)
2750_Down (R)
2750_NptⅠ (F)
NptⅠ_ApaⅠ (R)
5` 3`
Up stream
pmrB (A1S_2750)
Down stream
nptⅠ
Recombinant DNA
PCR using primers 2750_Up_SpeⅠ (F) and NptⅠ_ApaⅠ (R)
Up stream Down stream nptⅠ
Cloning sticky-ended PCR product into SpeⅠ and ApaⅠ – double digested pDM4
cat
pDM4 vector
sacB
Transformation by conjugation into A. baumannii ATCC17978 competent cell
chromosome
Up stream pmrB Down stream
First single cross-over homologus recombination
Up stream Down stream nptⅠ sacB cat Up stream pmrB Down stream
1
2
Second single cross-over homologous recombination excision
1) Wild-type strain
2) △A1S_2750 mutant strain

(C)

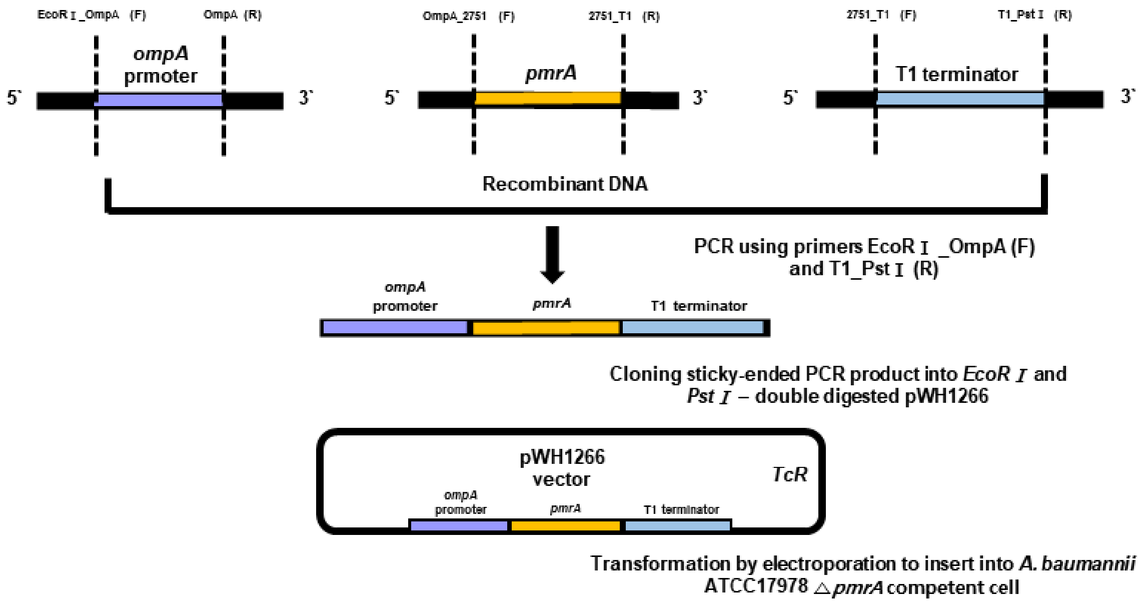

(D)

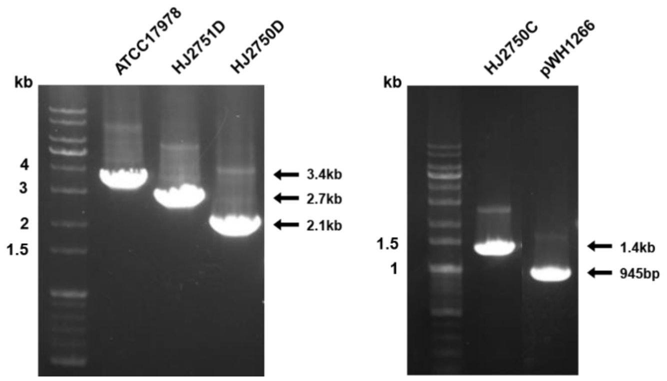

Supplementary Figure S1. Construction of the Δ*pmrA*, Δ*pmrB*, and *pmrA*-complemented strains. The schematic diagrams for constructing Δ*pmrA* (HJ2751D) (A), Δ*pmrB* (HJ2750D) mutant (B) strains, and *pmrA*-complemented strain (HJ2751C) (C). (D) Deletion of *pmrA* and *pmrB* in HJ2751D and HJ2750D using 2750_Up_SpeI (F) and 2751_Down (R) primers. The complementation of *pmrA* in HJ2751C using pWH1266 (F) and pWH1266 (R) primers (Supplementary Table S1). The amplicon size was 3,444 bp in the wild-type ATCC 17978, 2,775 bp in HJ2751D, 2,115 bp in HJ2750D, 1,442 bp in HJ2750C, and 945 bp in pWH1266 vector, respectively.
